# Texture Analysis of Routine Shoulder MRI for Predicting Postoperative Rotator Cuff Repair Integrity After Arthroscopic Rotator Cuff Repair

**DOI:** 10.7759/cureus.115547

**Published:** 2026-08-31

**Authors:** Keita Nagawa, Yuki Hara, Hirokazu Shimizu, Saki Tsuchihashi, Iichiro Osawa, Katsunobu Sakaguchi, Kaiji Inoue

**Affiliations:** 1 Radiology, Saitama Medical University Hospital, Moroyama, JPN; 2 Radiology, National Hospital Organization Takasaki Medical Center, Takasaki, JPN; 3 Radiology, Japanese Red Cross Ogawa Hospital, Ogawa, JPN; 4 Orthopedics, Saitama Medical University Hospital, Moroyama, JPN

**Keywords:** arthroscopic repair, machine learning, rotator cuff, shoulder, texture analysis

## Abstract

Introduction

Rotator cuff retear remains a major complication of arthroscopic rotator cuff repair (ARCR) and necessitates practical imaging-based methods for preoperative risk stratification. This study investigated whether texture features (TFs) extracted from routine shoulder MRI can predict postoperative tendon integrity, including the intermediate Sugaya type III category.

Methods

This retrospective study included 31 patients who had undergone ARCR. On preoperative shoulder MRI, the supraspinatus and infraspinatus muscles were manually segmented on a single oblique sagittal Y-view slice using proton density-weighted imaging (PDWI) and fat-suppressed T2-weighted imaging (FS-T2WI). Ninety-three TFs were extracted from each muscle in each sequence. Sequential feature selection (SFS) was applied, and classification models were developed using linear discriminant analysis (LDA), support vector machines (SVMs) with linear and radial basis function (RBF) kernels, and random forest (RF). Two binary classification tasks were evaluated: Sugaya types I-II versus III-V and Sugaya types I-II versus IV-V.

Results

For Sugaya types I-II versus III-V, the best single model used PDWI-derived supraspinatus features (accuracy, 0.792; receiver operating characteristic area under the curve (ROC AUC), 0.732). The best combination model achieved an accuracy of 0.815 and a ROC AUC of 0.833. For Sugaya types I-II versus IV-V, the best single model used the PDWI-derived combined supraspinatus and infraspinatus features (accuracy, 0.880; ROC AUC, 0.908). The best combination model achieved an accuracy of 0.839 and an ROC AUC of 0.864.

Conclusion

Texture analysis (TA) using routine preoperative shoulder MRI may help predict postoperative rotator cuff repair integrity, particularly the presence of definite postoperative tendon discontinuities.

## Introduction

Rotator cuff tears are among the most prevalent shoulder injuries. Arthroscopic rotator cuff repair (ARCR) is the standard of care owing to its favorable long-term outcomes [1]. Despite advances in surgical techniques, tendon retear remains a clinically significant complication that may compromise postoperative pain relief and functional recovery. The reported retear rates vary considerably across studies, and retears may occur even after an initially successful repair [2]. Although revision rotator cuff surgery is a treatment option for select patients, it is associated with higher complication rates and less favorable functional outcomes than primary repair [3,4]. These challenges underscore the importance of reliable preoperative risk stratification to guide surgical planning, patient counseling, and postoperative management.

Over the past several decades, numerous preoperative factors have been identified as predictors of retear, including tear size, tendon retraction, patient age, muscle atrophy, and fatty degeneration [5,6]. Among these, fatty degeneration and muscle atrophy are widely recognized as key indicators of poor tissue quality and reduced healing potential [7,8]. However, the conventional assessment methods have certain limitations. Qualitative grading systems, such as the Goutallier classification, are inherently subjective [9], whereas quantitative measures, including the fatty-infiltration ratio and degenerative fat area, frequently require specialized MRI techniques or dedicated imaging protocols that are not routinely acquired in standard clinical practice [10,11]. Therefore, a practical method that is capable of predicting postoperative retears using routinely acquired shoulder MRI sequences would have substantial clinical significance.

Texture analysis (TA) and radiomics have emerged as quantitative imaging techniques for extracting information beyond visual image assessments [12,13]. These approaches characterize the distribution and spatial organization of signal intensities within medical images and provide objective measures of tissue heterogeneity, uniformity, and microstructural complexity [12,13]. Such image-derived features may reflect underlying pathological changes, including collagen disorganization, edema, fibrosis, fatty degeneration, and other degenerative changes within the musculotendinous unit [14]. Consequently, texture features (TFs) may serve as imaging biomarkers of tissue quality and healing capacity in patients with rotator cuff tears.

Interest in this field has increased in recent years. Kang et al. [15] demonstrated that TFs extracted from torn supraspinatus tendons on preoperative T2-weighted magnetic resonance arthrography (MRA) were associated with postoperative tendon integrity. The retear group exhibited greater textural heterogeneity, characterized by higher entropy, variance, and contrast and lower angular second moment and inverse difference moment, suggesting that image-based textural heterogeneity may reflect impaired tendon healing potential. Recently, Fei et al. [16] developed a radiomics model for predicting postoperative rotator cuff tears using preoperative shoulder MRI. Based on three-dimensional volumes of interest encompassing the supraspinatus, infraspinatus, and humerus, the model demonstrated promising predictive performance. Furthermore, a nomogram integrating radiomic features with clinical variables improved the predictive discrimination. Collectively, these findings suggest that quantitative MRI analysis can provide valuable prognostic information regarding postoperative tendon integrity.

Nonetheless, despite these advances, several important issues remain unresolved. Previous studies have largely relied on a single MRI sequence or image type, which limits our understanding of whether predictive information can be robustly derived from multiple routinely acquired MRI sequences [15,16]. In addition, previous investigations have generally focused on dichotomized postoperative outcomes and have not explicitly addressed the intermediate postoperative tendon status represented by Sugaya classification type III [17], despite its relevance in routine clinical interpretation. Moreover, although recent radiomics studies have highlighted the potential of machine learning (ML)-based approaches, further research is required to determine their applicability in routine shoulder MRI protocols and clinically realistic classification frameworks.

Therefore, in the present study, we aimed to investigate whether TFs extracted from routine shoulder MRI could predict the risk of rotator cuff retear following ARCR. We specifically evaluated multiple routinely acquired MRI image types, applied ML-based predictive models, and assessed the postoperative tendon status using classification schemes that incorporated the intermediate Sugaya category. We hypothesized that TA on routine shoulder MRI would provide objective and clinically useful information for predicting postoperative tendon integrity and improving risk stratification beyond conventional imaging assessments alone.

## Materials and methods

Participants

This study was approved by the Research Ethics Committee of Saitama Medical University Hospital (approval number H2026-002). The requirement for informed consent was waived because of the retrospective study design. All procedures were conducted in accordance with the relevant guidelines and regulations.

We identified patients with rotator cuff tears who underwent shoulder MRI before ARCR (within three months of surgery) between January 2015 and October 2024. All MRI examinations were performed in the Department of Orthopedics at our institution.

The inclusion criteria were as follows: (i) age ≥15 years; (ii) a full-thickness rotator cuff tear identified on preoperative shoulder MRI; (iii) a full-thickness rotator cuff tear confirmed intraoperatively during ARCR performed at our institution; and (iv) postoperative shoulder MRI performed approximately 12 months after surgery (range, 11-13 months).

We initially identified 73 patients with rotator cuff tears who underwent shoulder MRI before ARCR within the study period. After applying the exclusion criteria - inadequate MRI data or absence of 12-month postoperative MRI (n = 9), preoperative MRI performed at another institution (n = 16), presence of MRI artifacts (n = 11), prior surgery on the affected shoulder (n = 2), and concomitant subscapularis tendon tear (n = 4) - a total of 31 patients were included in the final analysis.

Rotator cuff repair integrity was assessed using shoulder MRI performed 12 months postoperatively and classified according to the Sugaya classification [17] as follows: type I, sufficient tendon thickness with homogeneous low signal intensity; type II, sufficient tendon thickness with partial areas of high signal intensity; type III, insufficient tendon thickness (<50% of normal thickness) without discontinuity; type IV, presence of a minor discontinuity; and type V, presence of a major discontinuity. The classification of repair integrity was independently performed by two radiologists with 10 and 11 years of experience, respectively, who also performed the muscle delineation described below. Discrepancies were resolved by consensus, and the final classification was determined accordingly.

MRI acquisition and scanning parameters

MRI was performed using a 3.0-T scanner (Skyra; Siemens Healthcare, Erlangen, Germany) equipped with a dedicated shoulder coil. Among routine shoulder MRI sequences, oblique sagittal proton density-weighted imaging (PDWI) and oblique sagittal fat-suppressed T2-weighted imaging (FS-T2WI) were selected for subsequent analyses because both sequences were available for all patients.

The imaging parameters for oblique sagittal PDWI were as follows: repetition time, 1800 ms; echo time, 22 ms; flip angle, 90°; slice thickness, 3.5 mm; voxel size, 0.31 × 0.31 × 3.3 mm; and field of view, 14.0 cm. The imaging parameters for oblique sagittal FS-T2WI were as follows: repetition time, 2800 ms; echo time, 66 ms; flip angle, 90°; slice thickness, 3.5 mm; voxel size, 0.36 × 0.36 × 3.3 mm; and field of view, 14.0 cm.

Data analysis workflow

Figure 1 presents an overview of the workflow used in this study. To classify the postoperative tendon status while incorporating the intermediate category (Sugaya type III), two binary classification tasks were defined: (i) classification of Sugaya types I-II (sufficient tendon thickness) versus types III-V (insufficient tendon thickness or tendon discontinuity) and (ii) classification of Sugaya types I-II (sufficient tendon thickness) versus types IV-V (tendon discontinuity).

**Figure 1:**
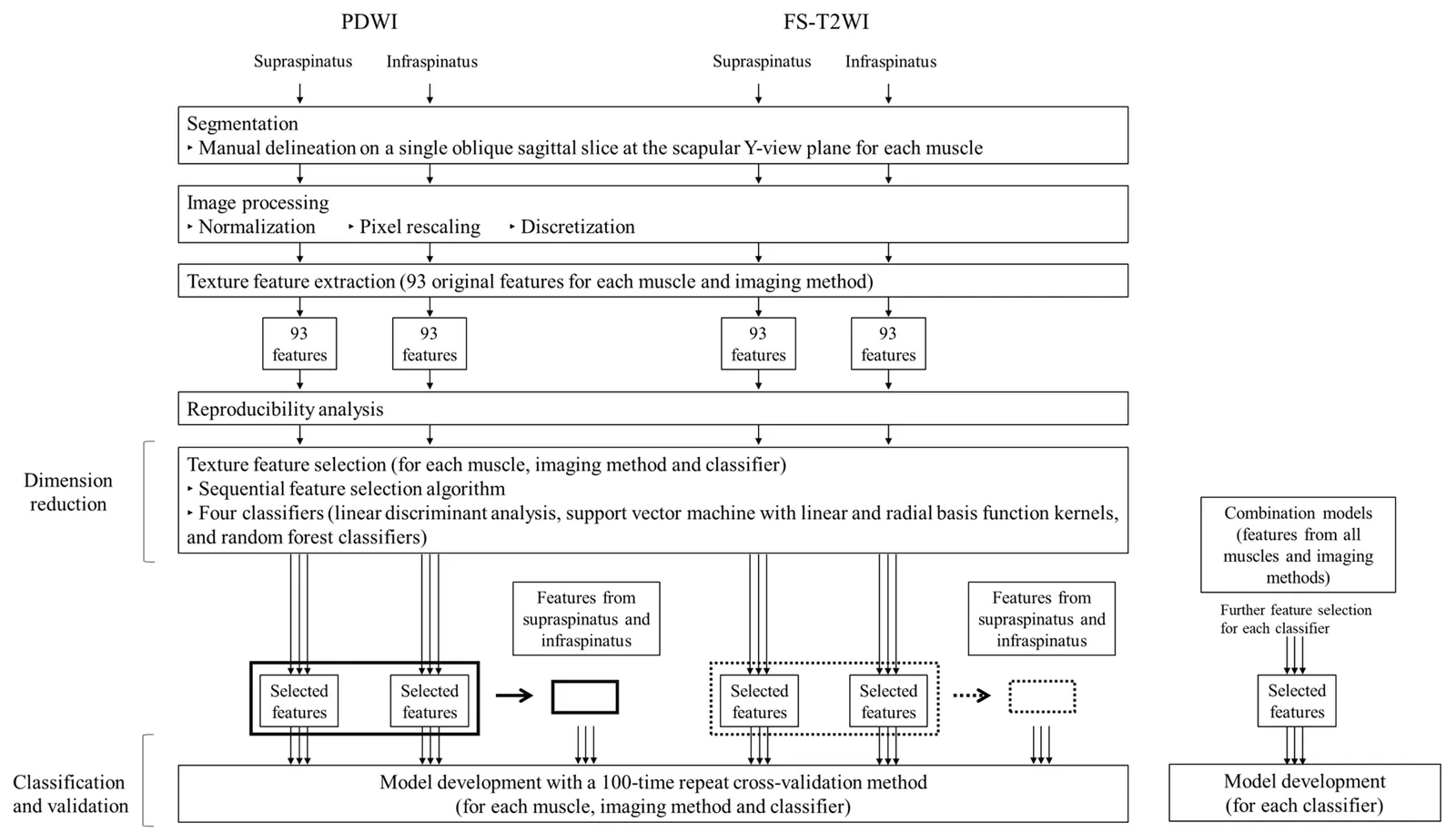
Overall study workflow Preoperative routine shoulder magnetic resonance imaging was used for manual segmentation of the supraspinatus and infraspinatus on oblique sagittal proton density-weighted imaging (PDWI) and fat-suppressed T2-weighted imaging (FS-T2WI). Following image preprocessing, textural features were extracted and subjected to reproducibility assessment and feature selection. Machine learning-based classification models were subsequently constructed and evaluated to predict the postoperative tendon status according to the Sugaya classification. Two binary classification tasks were designed: (i) classification of Sugaya types I-II (sufficient tendon thickness) versus types III-V (insufficient tendon thickness or tendon discontinuity) and (ii) classification of Sugaya types I-II (sufficient tendon thickness) versus types IV-V (tendon discontinuity).

Following the segmentation of the rotator cuff muscles, image preprocessing, TF extraction, and reproducibility analyses were performed separately for each rotator cuff muscle (i.e., the supraspinatus and infraspinatus), the combined muscle regions, and each imaging sequence (i.e., oblique sagittal PDWI and FS-T2WI). Subsequently, TF selection and ML-based model construction were performed independently for each classification task. In addition, combination models incorporating TFs derived from all muscle regions and imaging sequences were evaluated.

Segmentation and TF extraction

For muscle segmentation, MRI data were imported into the Digital Imaging and Communications in Medicine format using the open-source software package ITK-SNAP (version 3.8.0). The supraspinatus and infraspinatus were manually delineated on a single oblique sagittal slice in the scapular Y-view plane. To assess interobserver reproducibility, two radiologists with 10 and 11 years of experience, who were blinded to the clinical information, independently performed the segmentations. Figure 2 shows an example of the segmentation procedure.

**Figure 2:**
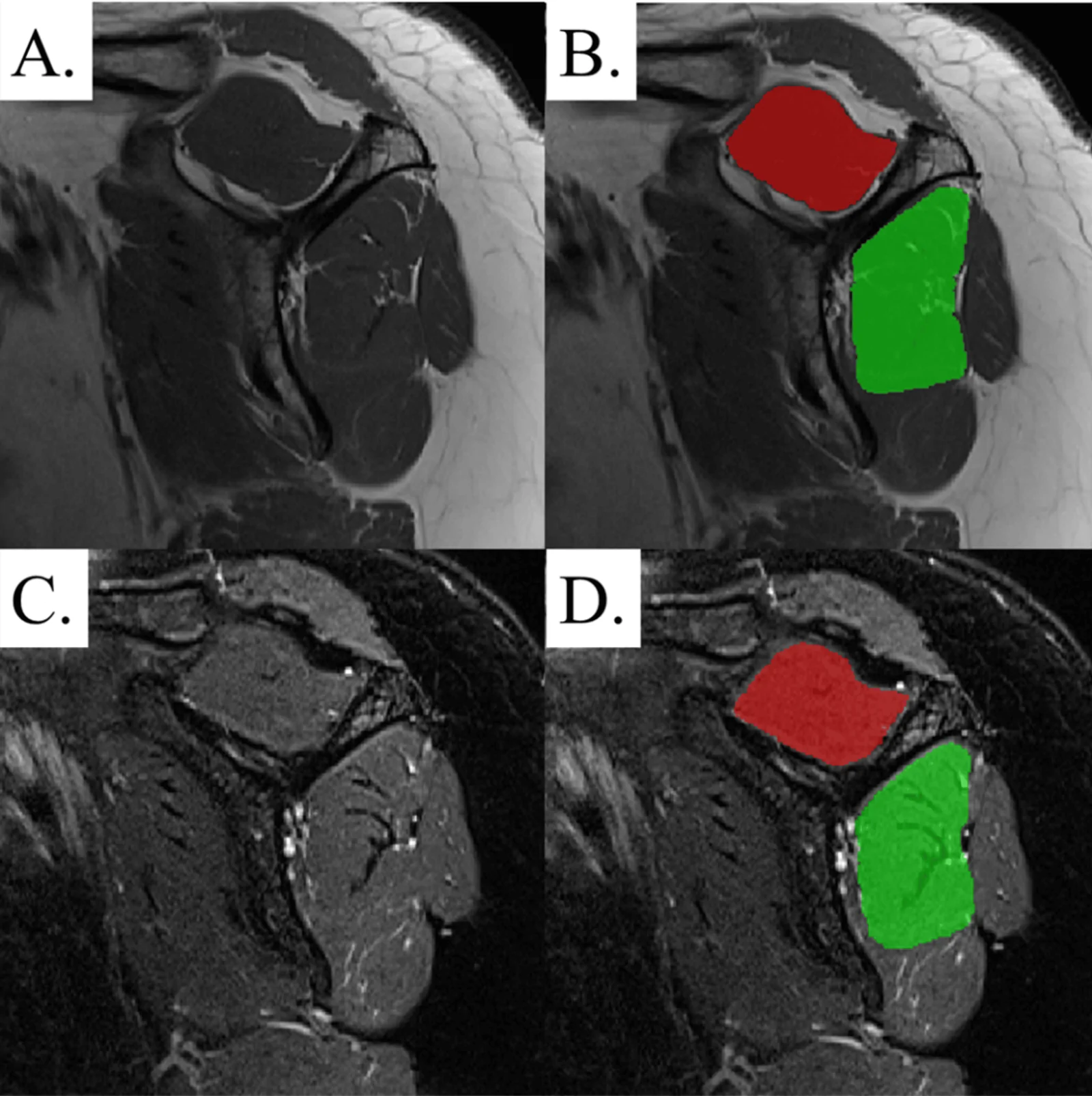
Representative examples of rotator cuff muscle segmentation on shoulder magnetic resonance imaging The upper row shows proton density-weighted images (A, B), and the lower row shows fat-suppressed T2-weighted images (C, D). The left columns (A, C) display the original images, whereas the right columns (B, D) show the corresponding contour delineations of the rotator cuff muscles. The supraspinatus and infraspinatus are outlined in red and green, respectively.

To minimize bias related to image heterogeneity, all MRI data underwent intensity normalization, with voxel intensities scaled to a range of 0-100, and were resampled to a uniform spatial resolution of 1 × 1 × 1 mm prior to feature extraction. The TFs were subsequently extracted using the open-source radiomics software package PyRadiomics (version 2.1.0), which enables the extraction of a comprehensive set of quantitative features from medical images. The extracted TFs were derived from six feature classes: first-order statistics, gray-level co-occurrence matrix (GLCM), gray-level dependence matrix (GLDM), gray-level run-length matrix (GLRLM), gray-level size zone matrix (GLSZM), and neighboring gray-tone difference matrix (NGTDM). A total of 93 TFs were extracted from each muscle for each imaging sequence and subsequently subjected to feature selection.

TF selection

Feature selection was performed prior to model construction to reduce dimensionality and minimize overfitting. Following the standardization of feature values to z-scores, the intraclass correlation coefficient (ICC) was calculated to assess interobserver reproducibility. Features with poor reproducibility (ICC < 0.7) were excluded from further analyses.

Feature selection was subsequently performed using a sequential feature selection (SFS) algorithm, which is a wrapper-based greedy search method that identifies subsets of features that maximize classification performance by iteratively adding or removing features according to a predefined classification model. We evaluated three representative classifiers: linear discriminant analysis (LDA), support vector machine (SVM), and random forest (RF). For the SVM, two kernel functions were tested separately: linear and radial basis function (RBF) kernels. Four classifiers were evaluated: LDA, SVM with a linear kernel, SVM with an RBF kernel, and RF. The subset of features that yielded the best classification accuracy was selected using the SFS algorithm. To reduce the risk of overfitting, the number of selected TFs was limited to three for each individual muscle- and sequence-based model and five for each combination model.

All preprocessing steps, including feature standardization (z-score normalization), reproducibility-based feature filtering (ICC < 0.7 exclusion), and SFS, were performed exclusively within the training data of each cross-validation fold. The held-out validation fold was not used in any preprocessing or feature-selection step. Parameters derived from each training fold were subsequently applied, without modification, to the corresponding validation fold to prevent information leakage.

Classification of Sugaya types I-II versus types III-V

Binary classification models were developed to distinguish Sugaya types I-II from Sugaya types III-V for each rotator cuff muscle and imaging sequence. Additionally, combined models incorporating both the supraspinatus and infraspinatus muscles were constructed. For each dataset, models were developed using LDA, an SVM with linear and RBF kernels, and RF classifiers. The model performance was evaluated using repeated 10-fold cross-validation with 100 repetitions to ensure the robustness and reproducibility of the results. Classifier performance was assessed using receiver operating characteristic (ROC) analysis and the area under the ROC curve (AUC). Accuracy, sensitivity, specificity, and F-measure were calculated.

Classification of Sugaya types I-II versus types IV-V

Binary classification models were also developed to distinguish Sugaya types I-II from types IV-V. Owing to class imbalance, the Synthetic Minority Oversampling Technique (SMOTE) was applied exclusively to the training data within each cross-validation iteration. SMOTE generates synthetic samples rather than exact duplicates, thereby increasing the size of the minority class, while preserving the underlying data distribution. Accordingly, the minority class is augmented, whereas the majority class remains unchanged. The validation fold was never included in the oversampling procedure and remained unmodified throughout model evaluation, thereby preventing information leakage between the training and validation datasets.

Classification models were developed using LDA and SVM with linear and RBF kernels, and RF classifiers. Model performance was evaluated using ROC analysis with repeated 10-fold cross-validation and 100 repetitions.

Combination models

In addition, combination models integrating TFs from all the rotator cuff muscles and imaging sequences were evaluated. Feature selection was performed using the SFS algorithm, with the number of selected TFs limited to five. Classification models were developed using the four ML classifiers described above, and their performance was evaluated using the same cross-validation framework and performance metrics.

All analyses were performed using the open-source Python package scikit-learn (version 0.22.1). Model performance metrics are reported as descriptive summaries (mean ± standard deviation across repeated cross-validation iterations); no formal statistical hypothesis tests were used to compare classifier or sequence performance.

## Results

A total of 31 shoulder MRI datasets from 31 patients were included in this study. The integrity of the postoperative rotator cuff repair was assessed using the Sugaya classification. Of the 31 patients, 16 were classified as type I-II (one as type I and 15 as type II), seven as type III, and eight as type IV-V (four as type IV and four as type V). Table 1 summarizes the demographic and clinical characteristics of the study population, stratified according to the Sugaya classification.

**Table 1:** Demographic and clinical characteristics of the study population

	Sugaya classification		
	Type I-II (n = 16)	Type III (n = 7)	Type IV-V (n = 8)
Age, years, mean ± SD	68.1 ± 6.0	65.1 ± 10.6	67.3 ± 9.9
Sex, male	9	4	3
Side, right	10	7	8
Symptom duration, months, mean ± SD	18.5 ± 21.8	33.0 ± 40.1	9.1 ± 7.0
Trauma onset	9	4	6
Tear size, n (%)			
Small	8 (50.0)	4 (57.1)	2 (25.0)
Medium	6 (37.5)	3 (42.9)	5 (62.5)
Large	2 (12.5)	0 (0.0)	1 (12.5)
Tear location, n (%)			
Supraspinatus	4 (25.0)	1 (14.3)	2 (25.0)
Supraspinatus and infraspinatus	12 (75.0)	6 (85.7)	6 (75.0)
Preoperative fatty infiltration (Goutallier and Fuchs criteria), mean ± SD	1.8 ± 0.8	1.6 ± 0.5	2.1 ± 0.6
Preoperative range of motion, deg, mean ± SD			
Forward flexion	166.3 ± 7.4	155.0 ± 18.9	137.1 ± 28.1
Abduction	158.7 ± 27.0	143.3 ± 28.1	127.1 ± 33.3

Interobserver reproducibility was good for all TFs. The mean ICC values ± standard deviation were 0.956 ± 0.038 and 0.940 ± 0.059 for the supraspinatus and infraspinatus on PDWI, respectively, and 0.965 ± 0.060 and 0.970 ± 0.044 for the supraspinatus and infraspinatus on FS-T2WI, respectively. Detailed ICC values for all TFs are provided in Appendix 1.

Classification between Sugaya types I-II and III-V

A subset of the three TFs was selected for each imaging sequence and muscle using the SFS algorithm. Table 2 lists the features selected for each classification task.

**Table 2:** Selected texture features in texture analysis-based models for classification of Sugaya types I-II versus types III-V This table summarizes the texture features selected by the sequential feature selection algorithm for each texture analysis-based classification model. Models were constructed using proton density-weighted imaging (PDWI) and fat-suppressed T2-weighted imaging (FS-T2WI) with features extracted from the supraspinatus, infraspinatus, and a combination of both muscles. The selected features are presented according to the classifier type. The feature classes included first-order statistics, gray-level co-occurrence matrix (GLCM), gray-level dependence matrix (GLDM), gray-level run-length matrix (GLRLM), gray-level size zone matrix (GLSZM), and neighboring gray-tone difference matrix (NGTDM). LDA: linear discriminant analysis; SVM: support vector machine; RBF: radial basis function; RF: random forest

		LDA	SVM with linear kernel	SVM with RBF kernel	RF
PDWI	Supraspinatus	Total energy (first-order), informational measure of correlation-2 (GLCM), and dependence entropy (GLDM)	Skewness (first-order), informational measure of correlation-2 (GLCM), and run entropy (GLRLM)	Mean (first-order), variance (first-order), and inverse variance (GLCM)	10th Percentile (first-order), difference entropy (GLCM), and inverse variance (GLCM)
	Infraspinatus	Minimum (first-order), correlation (GLCM), and long run high gray-level emphasis (GLRLM)	Correlation (GLCM), large dependence, high gray-level emphasis (GLDM), and gray-level non-uniformity normalized (GLRLM)	Inverse variance (GLCM), low gray-level emphasis (GLDM), and low gray-level run emphasis (GLRLM)	Small dependence, high gray-level emphasis (GLDM), long run high gray-level emphasis (GLRLM), and gray-level variance (GLSZM)
	Supraspinatus and infraspinatus	10th Percentile (first-order) and low gray-level emphasis (GLDM) from supraspinatus, and minimum (first-order), correlation (GLCM), and long run high gray-level emphasis (GLRLM) from infraspinatus	Entropy (first-order), informational measure of correlation-2 (GLCM) and dependence entropy (GLDM) from supraspinatus, and median (first-order) and correlation (GLCM) from infraspinatus	Interquartile range (first-order) and robust mean absolute deviation (first-order) from supraspinatus, inverse variance (GLCM), low gray-level emphasis (GLDM), and low gray-level run emphasis (GLRLM) from infraspinatus	90th percentile (first-order), maximum (first-order), inverse variance (GLCM), large area high gray-level emphasis (GLSZM), and contrast (NGTDM) from supraspinatus
FS-T2WI	Supraspinatus	10th percentile (first-order), total energy (first-order), and large area low gray-level emphasis (GLSZM)	Entropy (first-order), total energy (first-order), and long-run low gray-level emphasis (GLRLM)	Total energy (first-order), Joint average (GLCM), and long run low gray-level emphasis (GLRLM)	10th percentile (first-order), maximal correlation coefficient (GLCM), and small area low gray-level emphasis (GLSZM)
	Infraspinatus	Cluster tendency (GLCM), large dependence, high gray-level emphasis (GLDM), and gray-level non-uniformity normalized (GLSZM)	Entropy (first-order), maximum (first-order), and cluster shade (GLCM)	Skewness (first-order), maximal correlation coefficient (GLCM), and large area emphasis (GLSZM)	Minimum (first-order), difference entropy (GLCM), and informational measure of correlation-1 (GLCM)
	Supraspinatus and infraspinatus	10th percentile (first-order), total energy (first-order), and large area low gray-level emphasis (GLSZM) from supraspinatus, contrast (NGTDM), and gray-level non-uniformity normalized (GLSZM) from infraspinatus	Entropy (first-order), total energy (first-order), and long run low gray-level emphasis (GLRLM) from supraspinatus, and entropy (first-order) and cluster shade (GLCM) from infraspinatus	Total energy (first-order), joint average (GLCM), and long run low gray-level emphasis (GLRLM) from supraspinatus, and maximal correlation coefficient (GLCM) and large area emphasis (GLSZM) from infraspinatus	10th Percentile (first-order), maximal correlation coefficient (GLCM), and small area low gray-level emphasis from supraspinatus, and difference entropy (GLCM) and informational measure of correlation-1 (GLCM) from infraspinatus

Overall, the TA-based models demonstrated modest performance across all classification tasks. The best performance was achieved using PDWI of the supraspinatus with an SVM classifier employing an RBF kernel, which yielded an accuracy of 0.792 and an ROC AUC of 0.732. Table 3 summarizes the results of all the classification tasks, and Figure 3 shows the corresponding ROC curves. No substantial differences in classification performance were observed between the imaging sequences or muscles on descriptive comparison. Models yielding AUC values below 0.5 (e.g., PDWI Supraspinatus SVM-linear kernel: AUC, 0.244; Infraspinatus SVM-RBF kernel: AUC, 0.410; FS-T2WI Infraspinatus SVM-linear: AUC, 0.353) are likely attributable to a label-orientation inconsistency in the ROC computation and should not be interpreted as valid discriminative models. These results are reported for transparency but are excluded from the primary interpretation of model performance.

**Table 3:** Classification performance of texture analysis-based models for distinguishing Sugaya types I-II from types III-V Data presented are mean ± standard deviation. Classification models were developed using textural features extracted from proton density-weighted imaging (PDWI) and fat-suppressed T2-weighted imaging (FS-T2WI) of the supraspinatus and infraspinatus, and a combination of both muscles. The model performance was evaluated using accuracy, sensitivity, specificity, F-measure, and area under the receiver operating characteristic curve (ROC AUC). LDA: linear discriminant analysis; SVM: support vector machine; RBF: radial basis function; RF: random forest

			Accuracy	Sensitivity	Specificity	F-measure	ROC AUC
PDWI	Supraspinatus	LDA	0.630 ± 0.024	0.604 ± 0.030	0.655 ± 0.040	0.613 ± 0.024	0.655 ± 0.025
		SVM with linear kernel	0.602 ± 0.043	0.443 ± 0.074	0.751 ± 0.043	0.517 ± 0.067	0.244 ± 0.063
		SVM with RBF kernel	0.792 ± 0.024	0.667 ± 0.022	0.909 ± 0.046	0.757 ± 0.020	0.732 ± 0.027
		RF	0.613 ± 0.043	0.651 ± 0.074	0.578 ± 0.042	0.618 ± 0.051	0.654 ± 0.035
	Infraspinatus	LDA	0.700 ± 0.031	0.733 ± 0.046	0.669 ± 0.037	0.702 ± 0.033	0.685 ± 0.024
		SVM with linear kernel	0.695 ± 0.027	0.772 ± 0.033	0.623 ± 0.043	0.710 ± 0.025	0.669 ± 0.042
		SVM with RBF kernel	0.679 ± 0.041	0.471 ± 0.046	0.875 ± 0.063	0.587 ± 0.049	0.410 ± 0.102
		RF	0.455 ± 0.045	0.397 ± 0.079	0.509 ± 0.054	0.411 ± 0.068	0.483 ± 0.046
	Supraspinatus and infraspinatus	LDA	0.701 ± 0.032	0.735 ± 0.041	0.668 ± 0.039	0.704 ± 0.032	0.686 ± 0.019
		SVM with linear kernel	0.683 ± 0.030	0.687 ± 0.049	0.679 ± 0.035	0.677 ± 0.035	0.483 ± 0.084
		SVM with RBF kernel	0.679 ± 0.042	0.472 ± 0.055	0.873 ± 0.058	0.587 ± 0.056	0.415 ± 0.096
		RF	0.622 ± 0.054	0.618 ± 0.080	0.625 ± 0.067	0.611 ± 0.061	0.671 ± 0.029
FS-T2WI	Supraspinatus	LDA	0.629 ± 0.033	0.649 ± 0.049	0.610 ± 0.042	0.628 ± 0.036	0.670 ± 0.023
		SVM with linear kernel	0.731 ± 0.026	0.832 ± 0.051	0.636 ± 0.023	0.749 ± 0.029	0.730 ± 0.028
		SVM with RBF kernel	0.697 ± 0.042	0.751 ± 0.063	0.648 ± 0.049	0.705 ± 0.045	0.642 ± 0.073
		RF	0.674 ± 0.046	0.617 ± 0.073	0.728 ± 0.072	0.646 ± 0.062	0.688 ± 0.046
	Infraspinatus	LDA	0.569 ± 0.050	0.491 ± 0.068	0.643 ± 0.073	0.523 ± 0.058	0.585 ± 0.043
		SVM with linear kernel	0.628 ± 0.025	0.251 ± 0.034	0.981 ± 0.038	0.394 ± 0.047	0.353 ± 0.085
		SVM with RBF kernel	0.657 ± 0.052	0.539 ± 0.062	0.768 ± 0.065	0.603 ± 0.061	0.615 ± 0.073
		RF	0.545 ± 0.072	0.475 ± 0.089	0.612 ± 0.111	0.501 ± 0.078	0.547 ± 0.078
	Supraspinatus and infraspinatus	LDA	0.718 ± 0.029	0.728 ± 0.046	0.709 ± 0.038	0.714 ± 0.032	0.787 ± 0.020
		SVM with linear kernel	0.695 ± 0.031	0.756 ± 0.061	0.637 ± 0.033	0.705 ± 0.037	0.745 ± 0.037
		SVM with RBF kernel	0.677 ± 0.030	0.585 ± 0.041	0.763 ± 0.042	0.637 ± 0.036	0.609 ± 0.088
		RF	0.693 ± 0.057	0.677 ± 0.083	0.708 ± 0.078	0.680 ± 0.063	0.702 ± 0.045

**Figure 3:**
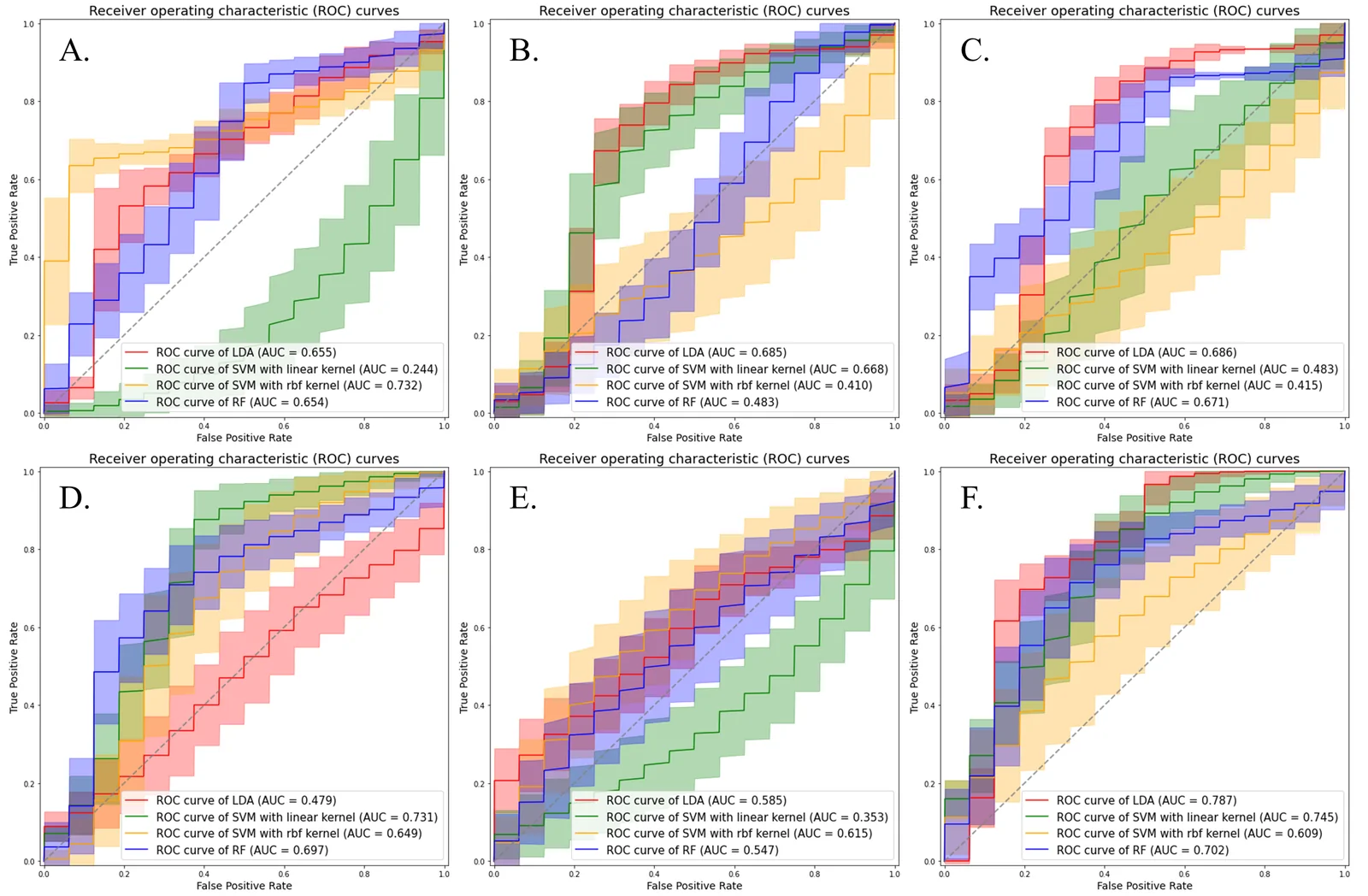
ROC curves of texture analysis-based classification models for differentiating Sugaya types I-II from types III-V Receiver operating characteristic (ROC) curves and area under the curve (AUC) values for classifying the postoperative tendon status between Sugaya types I-II and types III-V. Classification models were constructed using proton density-weighted imaging (A-C) and fat-suppressed T2-weighted imaging (D-F) based on texture features extracted from the supraspinatus (A, D), infraspinatus (B, E), and a combination of both muscles (C, F). LDA: linear discriminant analysis; RF: random forest; SVM: support vector machine

Combination models incorporating all imaging sequences and muscles achieved the best performance when the SVM classifier with a linear kernel was used, yielding an accuracy of 0.815 and an ROC AUC of 0.833. Table 4 presents the TFs selected for each classification task. Table 5 presents the classification results of the combination models.

**Table 4:** Selected texture features of combination models incorporating all imaging sequences and muscles for distinguishing Sugaya types I-II from types III-V This table summarizes the texture features selected for the combination models that incorporate all imaging sequences and both muscles. Models were constructed using proton density-weighted imaging (PDWI) and fat-suppressed T2-weighted imaging (FS-T2WI), with features extracted from the supraspinatus and infraspinatus. The selected features were presented according to the classifier type. The feature classes included first-order statistics, gray-level co-occurrence matrix (GLCM), gray-level dependence matrix (GLDM), gray-level run-length matrix (GLRLM), gray-level size zone matrix (GLSZM), and neighboring gray-tone difference matrix (NGTDM). LDA: linear discriminant analysis; SVM: support vector machine; RBF: radial basis function; RF: random forest

LDA		
PDWI	Infraspinatus	Long run emphasis (GLRLM)
FS-T2WI	Supraspinatus	10th Percentile (first-order)
FS-T2WI	Supraspinatus	Total energy (first-order)
FS-T2WI	Supraspinatus	Large area low gray-level emphasis (GLSZM)
FS-T2WI	Infraspinatus	Gray-level non-uniformity normalized (GLSZM)
SVM with linear kernel		
PDWI	Supraspinatus	Total energy (first-order)
PDWI	Supraspinatus	Cluster prominence (GLCM)
PDWI	Infraspinatus	Gray-level non-uniformity (GLRLM)
FS-T2WI	Supraspinatus	Entropy (first-order)
FS-T2WI	Supraspinatus	Long run low gray-level emphasis (GLRLM)
SVM with RBF kernel		
PDWI	Supraspinatus	90th Percentile (first-order)
PDWI	Supraspinatus	High gray-level zone emphasis (GLSZM)
FS-T2WI	Supraspinatus	Total energy (first-order)
FS-T2WI	Supraspinatus	Joint average (GLCM)
FS-T2WI	Supraspinatus	Long run low gray-level emphasis (GLRLM)
RF		
PDWI	Supraspinatus	Difference average (GLCM)
PDWI	Supraspinatus	Busyness (NGTDM)
FS-T2WI	Supraspinatus	Interquartile range (first-order)
FS-T2WI	Supraspinatus	High gray-level zone emphasis (GLSZM)
FS-T2WI	Supraspinatus	Small area low gray-level emphasis (GLSZM)

**Table 5:** Classification performance of combination models incorporating all imaging sequences and muscles for distinguishing Sugaya types I-II from types III-V Data presented are mean ± standard deviation. The combination models incorporated textural features extracted from proton density-weighted imaging (PDWI) and fat-suppressed T2-weighted imaging (FS-T2WI) of the supraspinatus and infraspinatus muscles. The model performance was evaluated using accuracy, sensitivity, specificity, F-measure, and area under the receiver operating characteristic curve (ROC AUC). LDA: linear discriminant analysis; SVM: support vector machine; RBF: radial basis function; RF: random forest

	Accuracy	Sensitivity	Specificity	F-measure	ROC AUC
LDA	0.639 ± 0.052	0.665 ± 0.079	0.616 ± 0.063	0.639 ± 0.060	0.627 ± 0.040
SVM with linear kernel	0.815 ± 0.040	0.847 ± 0.044	0.784 ± 0.062	0.816 ± 0.037	0.833 ± 0.032
SVM with RBF kernel	0.810 ± 0.033	0.711 ± 0.039	0.903 ± 0.049	0.783 ± 0.036	0.646 ± 0.074
RF	0.608 ± 0.052	0.641 ± 0.059	0.576 ± 0.084	0.613 ± 0.048	0.606 ± 0.040

Classification of Sugaya types I-II and IV-V

Table 6 lists the TFs selected for each classification task. The performance of the TA-based models ranged from modest to good, with models incorporating features derived from both the supraspinatus and infraspinatus generally outperforming those incorporating features derived from a single muscle. The best performance was achieved by the LDA model based on PDWI-derived features from both muscles, which yielded an accuracy of 0.880 and an ROC AUC of 0.908. Table 7 summarizes the results of all classification tasks, and Figure 4 presents the corresponding ROC curves.

**Table 6:** Selected texture features of texture analysis-based models for distinguishing Sugaya types I-II from types IV-V This table summarizes the texture features selected by the sequential feature selection algorithm for each texture analysis-based classification model. Models were constructed using proton density-weighted imaging (PDWI) and fat-suppressed T2-weighted imaging (FS-T2WI) with features extracted from the supraspinatus, infraspinatus, and a combination of both muscles. The selected features were presented according to the classifier type. The feature classes included first-order statistics, gray-level co-occurrence matrix (GLCM), gray-level dependence matrix (GLDM), gray-level run-length matrix (GLRLM), gray-level size zone matrix (GLSZM), and neighboring gray-tone difference matrix (NGTDM). LDA: linear discriminant analysis; SVM: support vector machine; RBF: radial basis function; RF: random forest

		LDA	SVM with linear kernel	SVM with RBF kernel	RF
PDWI	Supraspinatus	90th Percentile (first-order), total energy (first-order), and difference variance (GLCM)	10th Percentile (first-order), kurtosis (first-order), and root mean squared (first-order)	Correlation (GLCM), informational measure of correlation-1 (GLCM), and zone entropy (GLSZM)	Root mean squared (first-order), inverse variance (GLCM), and gray-level variance (GLDM)
	Infraspinatus	10th Percentile (first-order), cluster shade (GLCM), and long run low gray-level emphasis (GLRLM)	10th Percentile (first-order), large area low gray-level emphasis (GLSZM) and contrast (NGTDM)	90th Percentile (first-order), median (first-order), small dependence, high gray-level emphasis (GLDM)	Inverse difference moment normalized (GLCM), large dependence low gray-level emphasis (GLDM), and long run low gray-level emphasis (GLRLM)
	Supraspinatus and infraspinatus	Kurtosis (first-order) and median (first-order) from supraspinatus, and cluster shade (GLCM), long run low gray-level emphasis (GLRLM), and large area high gray-level emphasis (GLSZM) from infraspinatus	10th Percentile (first-order), entropy (first-order), and mean (first-order) from supraspinatus, and large area low gray-level emphasis (GLSZM) and contrast (NGTDM) from infraspinatus	Correlation (GLCM), maximal correlation coefficient (GLCM), and zone entropy (GLSZM) from supraspinatus, and median (first-order) and long run high gray-level emphasis (GLRLM) from infraspinatus	10th Percentile (first-order), inverse variance (GLCM), and high gray-level emphasis (GLDM) from supraspinatus, and 10th Percentile (first-order) and low gray-level emphasis (GLDM) from infraspinatus
FS-T2WI	Supraspinatus	10th Percentile (first-order), minimum (first-order), and gray-level variance (GLRLM)	Energy (first-order), dependence variance (GLDM), and contrast (NGTDM)	Robust mean absolute deviation (first-order), difference entropy (GLCM), and small area low gray-level emphasis (GLSZM)	Minimum (first-order), inverse difference normalized (GLCM), and low gray-level zone emphasis (GLSZM)
	Infraspinatus	10th Percentile (first-order), mean absolute deviation (first-order), and contrast (GLCM)	90th Percentile (first-order), total energy (first-order), and cluster prominence (GLCM)	10th Percentile (first-order), dependence variance (GLDM), and contrast (NGTDM)	Interquartile range (first-order), inverse difference moment normalized (GLCM), and dependence variance (GLDM)
	Supraspinatus and infraspinatus	10th Percentile (first-order), minimum (first-order), and gray-level variance (GLRLM) from supraspinatus, and 10th Percentile (first-order) and minimum (first-order) from infraspinatus	Energy (first-order), minimum (first-order), and contrast (NGTDM) from supraspinatus, and large area low gray-level emphasis (GLSZM) and strength (NGTDM) from infraspinatus	Minimum (first-order) and autocorrelation (GLCM) from supraspinatus, and 10th percentile (first-order), large area emphasis (GLSZM), and large area low gray-level emphasis (GLSZM) from infraspinatus	Minimum (first-order), inverse difference normalized (GLCM), large dependence low gray-level emphasis (GLDM), and contrast (NGTDM) from supraspinatus, and coarseness (NGTDM) from infraspinatus

**Table 7:** Classification performance of texture analysis-based models for distinguishing Sugaya types I-II from types IV-V Data presented are mean ± standard deviation. Classification models were developed using textural features extracted from proton density-weighted imaging (PDWI) and fat-suppressed T2-weighted imaging (FS-T2WI) of the supraspinatus and infraspinatus, and a combination of both muscles. The model performance was evaluated using accuracy, sensitivity, specificity, F-measure, and area under the receiver operating characteristic curve (ROC AUC). LDA: linear discriminant analysis; SVM: support vector machine; RBF: radial basis function; RF: random forest

			Accuracy	Sensitivity	Specificity	F-measure	ROC AUC
PDWI	Supraspinatus	LDA	0.683 ± 0.027	0.731 ± 0.038	0.636 ± 0.042	0.698 ± 0.027	0.710 ± 0.025
		SVM with linear kernel	0.584 ± 0.037	0.664 ± 0.056	0.504 ± 0.057	0.614 ± 0.038	0.624 ± 0.037
		SVM with RBF kernel	0.642 ± 0.041	0.523 ± 0.030	0.761 ± 0.071	0.594 ± 0.037	0.671 ± 0.048
		RF	0.764 ± 0.030	0.849 ± 0.046	0.679 ± 0.052	0.782 ± 0.028	0.886 ± 0.016
	Infraspinatus	LDA	0.714 ± 0.016	0.626 ± 0.006	0.803 ± 0.031	0.687 ± 0.012	0.767 ± 0.019
		SVM with linear kernel	0.760 ± 0.032	0.628 ± 0.045	0.893 ± 0.047	0.723 ± 0.038	0.783 ± 0.027
		SVM with RBF kernel	0.676 ± 0.028	0.563 ± 0.006	0.789 ± 0.055	0.635 ± 0.020	0.609 ± 0.040
		RF	0.553 ± 0.054	0.634 ± 0.075	0.472 ± 0.071	0.586 ± 0.055	0.564 ± 0.049
	Supraspinatus and infraspinatus	LDA	0.880 ± 0.016	0.875 ± 0.010	0.885 ± 0.032	0.880 ± 0.014	0.908 ± 0.008
		SVM with linear kernel	0.783 ± 0.022	0.647 ± 0.030	0.920 ± 0.029	0.749 ± 0.026	0.756 ± 0.023
		SVM with RBF kernel	0.691 ± 0.037	0.661 ± 0.049	0.720 ± 0.060	0.681 ± 0.038	0.749 ± 0.029
		RF	0.832 ± 0.032	0.924 ± 0.030	0.740 ± 0.054	0.846 ± 0.028	0.952 ± 0.014
FST2WI	Supraspinatus	LDA	0.740 ± 0.035	0.711 ± 0.050	0.770 ± 0.043	0.732 ± 0.038	0.820 ± 0.016
		SVM with linear kernel	0.581 ± 0.024	0.403 ± 0.036	0.759 ± 0.045	0.489 ± 0.031	0.449 ± 0.071
		SVM with RBF kernel	0.381 ± 0.022	0.267 ± 0.056	0.494 ± 0.057	0.299 ± 0.049	0.594 ± 0.038
		RF	0.724 ± 0.034	0.771 ± 0.039	0.677 ± 0.064	0.737 ± 0.029	0.839 ± 0.025
	Infraspinatus	LDA	0.781 ± 0.031	0.750 ± 0.010	0.8125 ± 0.0625	0.775 ± 0.025	0.791 ± 0.011
		SVM with linear kernel	0.546 ± 0.048	0.516 ± 0.079	0.576 ± 0.057	0.530 ± 0.062	0.491 ± 0.088
		SVM with RBF kernel	0.860 ± 0.025	0.809 ± 0.028	0.911 ± 0.038	0.853 ± 0.025	0.884 ± 0.012
		RF	0.727 ± 0.019	0.634 ± 0.027	0.819 ± 0.023	0.698 ± 0.023	0.703 ± 0.029
	Supraspinatus and infraspinatus	LDA	0.768 ± 0.028	0.788 ± 0.031	0.749 ± 0.045	0.773 ± 0.026	0.801 ± 0.014
		SVM with linear kernel	0.582 ± 0.036	0.228 ± 0.043	0.936 ± 0.058	0.351 ± 0.059	0.330 ± 0.073
		SVM with RBF kernel	0.868 ± 0.033	0.851 ± 0.054	0.885 ± 0.044	0.865 ± 0.035	0.910 ± 0.022
		RF	0.627 ± 0.047	0.643 ± 0.063	0.610 ± 0.069	0.632 ± 0.048	0.698 ± 0.033

**Figure 4:**
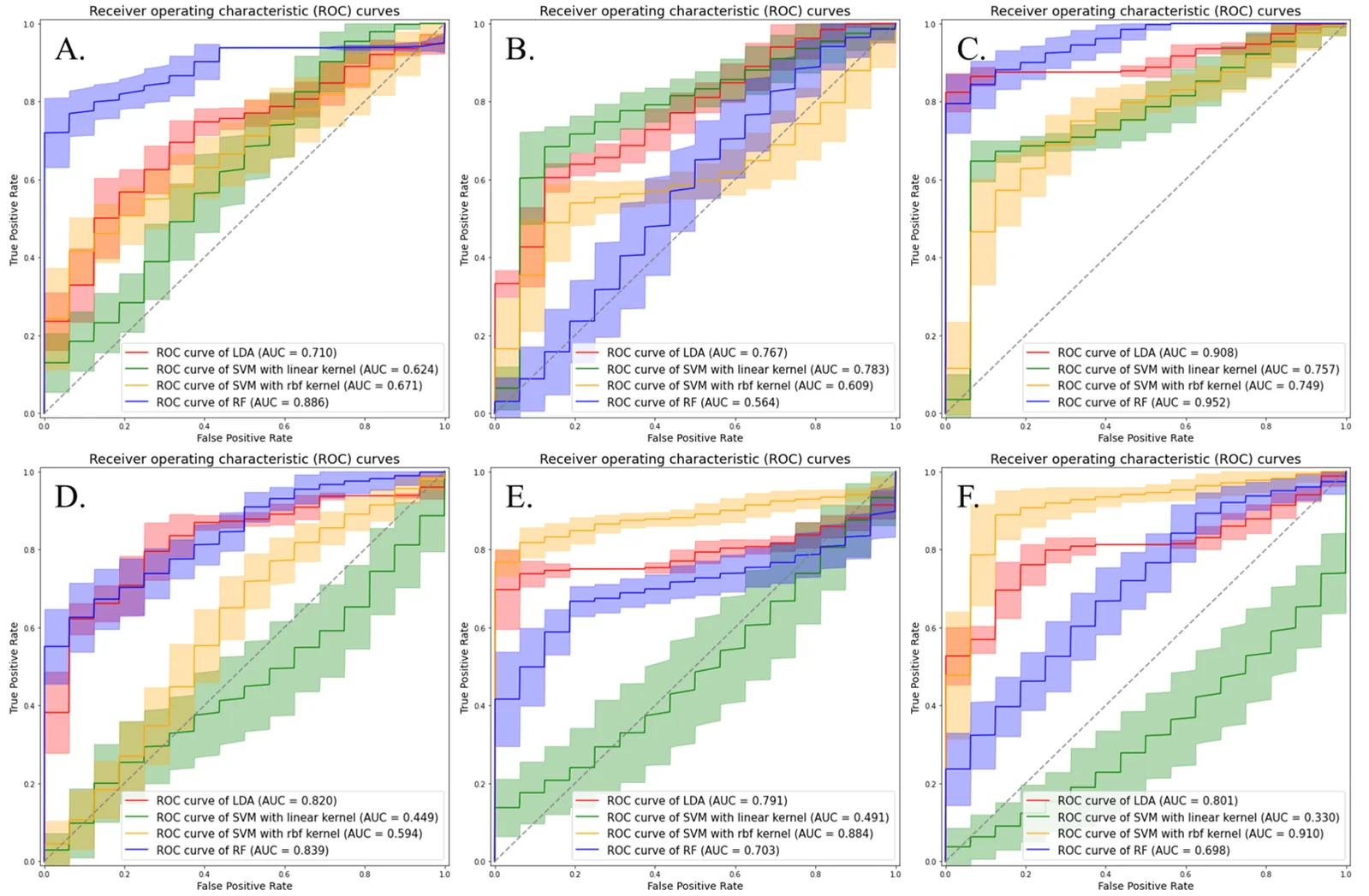
ROC curves of texture analysis-based classification models for differentiating Sugaya types I-II from types IV-V Receiver operating characteristic (ROC) curves and area under the curve (AUC) values for classifying postoperative tendon status as Sugaya types I-II and IV-V. Models were constructed using proton density-weighted imaging (A-C) and fat-suppressed T2-weighted imaging (D-F) based on texture features extracted from the supraspinatus (A, D), infraspinatus (B, E), and a combination of both muscles (C, F). LDA: linear discriminant analysis; RF: random forest; SVM: support vector machine

The combination models incorporating all imaging sequences and muscles achieved the highest performance with LDA, yielding an accuracy of 0.839 and an ROC AUC of 0.864. Table 8 presents the TFs selected for each classification task. Table 9 presents the classification results of the combination models.

**Table 8:** Selected texture features of combination models incorporating all imaging sequences and muscles for distinguishing Sugaya types I-II from types IV-V This table summarizes the texture features selected for the combination models that incorporate all imaging sequences and both muscles. Models were constructed using proton density-weighted imaging (PDWI) and fat-suppressed T2-weighted imaging (FS-T2WI), with features extracted from the supraspinatus and infraspinatus. The selected features were presented according to the classifier type. The feature classes included first-order statistics, gray-level co-occurrence matrix (GLCM), gray-level dependence matrix (GLDM), gray-level run-length matrix (GLRLM), gray-level size zone matrix (GLSZM), and neighboring gray-tone difference matrix (NGTDM). LDA: linear discriminant analysis; SVM: support vector machine; RBF: radial basis function; RF: random forest

LDA		
PDWI	Supraspinatus	Kurtosis (first-order)
PDWI	Supraspinatus	Median (first-order)
PDWI	Infraspinatus	Cluster shade (GLCM)
PDWI	Infraspinatus	Long run low gray-level emphasis (GLRLM)
PDWI	Infraspinatus	Large area high gray-level emphasis (GLSZM)
SVM with linear kernel		
PDWI	Supraspinatus	10th Percentile (first-order)
PDWI	Supraspinatus	Entropy (first-order)
PDWI	Supraspinatus	Mean (first-order)
PDWI	Infraspinatus	Large area low gray-level emphasis (GLSZM)
PDWI	Infraspinatus	Contrast (NGTDM)
SVM with RBF kernel		
PDWI	Supraspinatus	Energy (first-order)
PDWI	Supraspinatus	Correlation (GLCM)
PDWI	Supraspinatus	Informational measure of correlation-1 (GLCM)
PDWI	Supraspinatus	Zone entropy (GLSZM)
FS-T2WI	Infraspinatus	High gray-level zone emphasis (GLSZM)
RF		
PDWI	Supraspinatus	Inverse difference moment normalized (GLCM)
PDWI	Supraspinatus	Inverse variance (GLCM)
PDWI	Supraspinatus	Short run high gray level emphasis (GLRLM)
PDWI	Infraspinatus	Low gray-level run emphasis (GLRLM)
PDWI	Infraspinatus	Run length non-uniformity (GLRLM)

**Table 9:** Classification performance of combination models incorporating all imaging sequences and muscles for distinguishing Sugaya types I-II from types IV-V Data presented are mean ± standard deviation. The combination models incorporated textural features extracted from proton density-weighted imaging (PDWI) and fat-suppressed T2-weighted imaging (FS-T2WI) of the supraspinatus and infraspinatus muscles. The model performance was evaluated using accuracy, sensitivity, specificity, F-measure, and area under the receiver operating characteristic curve (ROC AUC). LDA: linear discriminant analysis; SVM: support vector machine; RBF: radial basis function; RF: random forest

	Accuracy	Sensitivity	Specificity	F-measure	ROC AUC
LDA	0.839 ± 0.025	0.793 ± 0.038	0.885 ± 0.032	0.831 ± 0.029	0.864 ± 0.029
SVM with linear kernel	0.650 ± 0.025	0.463 ± 0.032	0.837 ± 0.045	0.569 ± 0.030	0.659 ± 0.061
SVM with RBF kernel	0.675 ± 0.026	0.564 ± 0.009	0.787 ± 0.051	0.635 ± 0.019	0.690 ± 0.028
RF	0.710 ± 0.037	0.771 ± 0.052	0.648 ± 0.058	0.726 ± 0.036	0.870 ± 0.023

## Discussion

In the present study, we investigated whether TFs extracted from routine preoperative shoulder MRI scans could predict postoperative tendon integrity after ARCR. The predictive performance of our TA-based models was modest for distinguishing Sugaya types I-II from types III-V, and modest to good for distinguishing Sugaya types I-II from types IV-V. These preliminary findings suggest that TFs derived from routinely acquired preoperative shoulder MRI may have potential for postoperative tendon-integrity risk stratification without requiring dedicated imaging sequences. However, given the small single-centre cohort and absence of external validation, the present models should be regarded as exploratory and hypothesis-generating and should not yet be used for clinical decision-making. Validation in larger independent multicentre cohorts is required before clinical applicability can be established.

Our findings should be interpreted in the context of previous studies that applied quantitative image analysis to predict the postoperative tendon status. Kang et al. [15] demonstrated that the textural features extracted from the torn supraspinatus tendon on preoperative T2-weighted MRA were associated with postoperative tendon integrity after repair. Specifically, the retear group exhibited higher entropy, variance, and contrast and a lower angular second moment and inverse difference moment, indicating greater textural heterogeneity in tendons that subsequently failed after repair [15]. More recently, Fei et al. [16] developed a radiomics model for predicting postoperative rotator cuff re-tear using preoperative MRI and reported favorable performance, particularly when radiomic features from multiple regions were combined and integrated with clinical variables. Their findings further suggested that quantitative MRI-based analysis may provide prognostic information beyond that obtained from conventional imaging interpretations [16]. This study extends these previous reports in several ways. Unlike Kang et al. [15], who analyzed the torn tendon itself on MRA, we evaluated the rotator cuff muscles using routine non-arthrographic MRI, a modality that is more widely available in clinical practice. Compared to Fei et al. [16], who performed three-dimensional radiomics analysis of the supraspinatus, infraspinatus, and humerus on oblique coronal fat-suppressed T2-weighted images, our study focused on TFs derived from routine oblique sagittal PDWI and FS-T2WI obtained in the Y-view plane. Furthermore, whereas previous studies primarily examined dichotomized outcomes, we evaluated a clinically relevant classification task that included the intermediate postoperative tendon status, represented by Sugaya type III. Although these methodological differences may have increased the complexity of the prediction task, they also enhanced the applicability of our approach to routine clinical MRI assessments.

The present findings are consistent with those of Kang et al. [15], who identified entropy, variance, and contrast as informative TFs for predicting postoperative tendon status after rotator cuff repair. Despite substantial differences in the target tissue, MRI protocol, and analytical framework, the repeated identification of these features suggests that they may capture the fundamental aspects of tissue degeneration associated with impaired postoperative healing [18]. From an imaging perspective, entropy reflects signal randomness or disorder, variance reflects the dispersion of signal intensities, and contrast reflects the local intensity differences between neighboring pixels [19]. Higher values of these parameters generally indicate greater tissue heterogeneity [19], which may correspond to degeneration-related changes such as fatty infiltration, fibrosis, architectural disorganization, or chronic alterations of the musculotendinous unit [14]. Therefore, the concordance between our findings and those of Kang et al. [15] supports the hypothesis that textural heterogeneity is a meaningful imaging biomarker of impaired healing capacity rather than a statistical artifact.

In our results, several first-order and higher-order TFs were selected in the classification models, including first-order metrics such as the 10th percentile, 90th percentile, and total energy, as well as higher-order features derived from the GLRLM and GLSZM. This broad pattern of feature selection suggests that the imaging signature associated with postoperative healing failure is multidimensional. First-order TFs describe the overall distribution and magnitude of voxel intensities, whereas higher-order TFs provide additional information regarding spatial organization, including repeated intensity patterns and the size of homogeneous signal regions [20-22]. These features may reflect patchy fatty degeneration, fibrosis, or regional architectural disorganization within rotator cuff muscles. Thus, postoperative tendon integrity may be associated not only with overall signal heterogeneity, but also with the distribution, clustering, and spatial complexity of abnormalities within the musculotendinous unit.

Our results share conceptual similarities with those reported by Fei et al. [16]. In their study, a prognostic radiomics model incorporating multiple regions outperformed models based on a single region, and the infraspinatus contributed substantially to prediction performance [16]. Similarly, in the present study, models incorporating both the supraspinatus and infraspinatus muscles generally performed better than single-muscle models, and combination models using multiple muscles and sequences demonstrated favorable performance. These findings support the concept that postoperative tendon integrity is influenced not only by the local condition of the torn tendon or a single muscle but also by the broader structural and functional status of the rotator cuff unit. Although primary tears often involve the supraspinatus, the infraspinatus may influence load-sharing, force coupling, tendon mobility, repair tension, and healing potential [23]. Therefore, a comprehensive assessment of the rotator cuff musculature may better capture the biological environment in which tendon healing occurs.

However, the predictive performance observed in our study was modest compared with that reported in previous radiomic studies. Several factors may explain this discrepancy. First, our classification task was more challenging because we explicitly included Sugaya type III in one analysis. Sugaya type III represents an intermediate postoperative tendon morphology without definite discontinuity [17] and may be biologically and radiologically closer to the intact group than to the definite retear group, making discrimination inherently more difficult. This likely explains why the TA-based models performed better when distinguishing Sugaya types I-II from IV-V than when distinguishing Sugaya types I-II from III-V. Second, unlike Fei et al. [16], who used three-dimensional volumetric segmentation and incorporated humeral features and clinical factors into a nomogram, our analysis was based on a single oblique sagittal slice and imaging-derived TFs alone. Although this simplified approach improves the practicality and reproducibility, it may reduce the amount of structural information available to the model. Third, our use of routine clinical MRI sequences, which are advantageous for clinical applicability, may provide less specialized contrast for tissue characterization than dedicated radiomics-oriented imaging protocols.

Notably, for the classification of Sugaya types I-II versus IV-V, the single-sequence dual-muscle PDWI model (LDA: accuracy 0.880, AUC 0.908) outperformed the combination model integrating all sequences and muscles (LDA: accuracy 0.839, AUC 0.864). This seemingly counterintuitive finding may reflect the well-known 'curse of dimensionality' in small-cohort settings: adding features from additional sequences, even after selection, may introduce noise and reduce model stability when the training sample size is severely limited. This observation underscores the importance of carefully matching model complexity to the available sample size and suggests that, in small-cohort radiomics research, simpler models may generalize better than more complex multi-source alternatives.

No substantial differences were observed between the imaging sequences or muscles in the individual models, although PDWI tended to yield the best-performing single-sequence models. One possible explanation is that PDWI provides a relatively stable depiction of intramuscular architecture and signal distribution [24], facilitating the extraction of TFs related to subtle chronic structural degeneration. FS-T2WI may additionally capture fluid-sensitive changes such as edema or inflammatory alterations [25]; however, these signal characteristics may be more variable and less specific for chronic tissue quality. Nevertheless, the comparable performance of PDWI and FS-T2WI supports the potential robustness of TA across routine shoulder MRI protocols, representing an important clinical advantage.

From a clinical perspective, if the risk of postoperative structural failure can be estimated using routine preoperative MRI, this information may help refine surgical planning, improve patient counseling, and support individualized postoperative management. For example, patients with a higher risk of healing failure may benefit from closer postoperative imaging surveillance, more cautious rehabilitation progression, or consideration of adjunctive surgical strategies. Fei et al. [16] similarly emphasized that radiomics models may help guide individualized decision-making. Our findings support this concept by suggesting that clinically relevant information can be extracted from standard routine MRI sequences. Although the current predictive performance is insufficient for stand-alone clinical decision-making, TA-based models may serve as complementary tools within a multiparametric framework incorporating morphological MRI findings and clinical variables.

This study had some limitations. First, the sample size was small, which may have reduced the statistical power and affected model stability despite repeated cross-validation. Second, this was a retrospective single-center study; therefore, selection bias could not be excluded. Third, segmentation was performed on a single oblique sagittal slice in the Y-view plane rather than using full three-dimensional muscle volumes. Fourth, postoperative tendon status was assessed using MRI according to the Sugaya classification and was not confirmed surgically. Fifth, we focused primarily on imaging-derived TFs and did not incorporate other potentially relevant clinical or morphological variables, such as tear size, tendon retraction, repair configuration, rehabilitation protocol, or comorbidities, into the models. Given the favorable performance of the combined radiomics-clinical nomogram reported by Fei et al. [16], incorporating these variables may further enhance the predictive accuracy in future studies. Sixth, a label-orientation inconsistency was identified in the ROC computation for several models, resulting in AUC values below 0.5. This technical issue, which could not be fully resolved due to software pipeline constraints, affects the reliability of the implicated models and limits the conclusions that can be drawn from them. Future studies should ensure consistent positive class label definition across all models prior to ROC analysis. Finally, external validation was not performed; thus, the generalizability of our findings remains uncertain.

Future studies should include larger multicenter cohorts, external validation, and volumetric segmentation to determine whether the predictive performance can be improved while maintaining clinical applicability. The development of hybrid models combining TFs with conventional clinical and morphological predictors may improve risk stratification. Further research is needed to clarify the biological correlates of selected features, particularly whether entropy-, variance-, and contrast-related TFs, as well as other first- and higher-order radiomic features, reflect specific histopathological changes, such as fatty degeneration, fibrosis, collagen disorganization, or alterations in muscle quality.

## Conclusions

The TA of the supraspinatus and infraspinatus muscles on routine preoperative shoulder MRI may provide useful information for predicting the integrity of postoperative rotator cuff repair. Although the TA-based models demonstrated a modest overall performance, better results were observed for the classification task targeting definite tendon discontinuities. By extending previous tendon- and radiomics-based studies to routine non-arthrographic MRI and clinically relevant classification settings, our findings support the potential role of MRI-based quantitative analysis as a complementary approach to retear risk stratification after ARCR. Given the small single-centre cohort and absence of external validation, the present models should be regarded as exploratory and hypothesis-generating and should not yet be used for clinical decision-making. Validation in larger independent multicentre cohorts is required before clinical applicability can be established.

## Appendices

Appendix 1

**Table 10:** Interobserver reproducibility test for each texture feature subset The intra-class correlation coefficient was calculated to evaluate interobserver reproducibility. FS-PDWI, fat-suppressed proton density-weighted imaging; GLCM, gray-level co-occurrence matrix; GLDM, gray-level dependence matrix; GLRLM, gray-level run-length matrix; GLSZM, gray-level size zone matrix; NGTDM, neighboring grey-tone difference matrix; PDWI, proton density-weighted imaging

	PDWI		FS-T2WI	
	Supraspinatus	Infraspinatus	Supraspinatus	Infraspinatus
10th Percentile (first-order)	0.990	0.985	0.979	0.964
90th Percentile (first-order)	0.983	0.983	0.986	0.989
Energy (first-order)	0.990	0.977	0.986	0.988
Entropy (first-order)	0.982	0.982	0.998	0.991
Interquartile range (first-order)	0.964	0.957	0.999	0.996
Kurtosis (first-order)	0.783	0.829	0.717	0.828
Maximum (first-order)	0.898	0.758	0.842	0.910
Mean absolute deviation (first-order)	0.974	0.972	0.999	0.996
Mean (first-order)	0.988	0.989	0.985	0.982
Median (first-order)	0.986	0.987	0.999	0.995
Minimum (first-order)	0.912	0.914	0.849	0.968
Range (first-order)	0.876	0.791	0.826	0.910
Robust mean absolute deviation (first-order)	0.969	0.964	0.999	0.986
Root mean squared (first-order)	0.987	0.988	0.993	0.992
Skewness (first-order)	0.860	0.801	0.956	0.991
Total energy (first-order)	0.983	0.959	0.972	0.952
Uniformity (first-order)	0.977	0.979	1.000	0.972
Variance (first-order)	0.970	0.965	0.974	0.978
Autocorrelation (GLCM)	0.959	0.932	0.980	0.985
Cluster prominence (GLCM)	0.928	0.911	0.774	0.795
Cluster shade (GLCM)	0.844	0.893	0.735	0.779
Cluster tendency (GLCM)	0.973	0.966	0.971	0.977
Contrast (GLCM)	0.984	0.964	0.970	0.987
Correlation (GLCM)	0.978	0.964	1.000	0.999
Difference average (GLCM)	0.987	0.978	0.986	0.990
Difference entropy (GLCM)	0.986	0.981	0.999	0.986
Difference variance (GLCM)	0.975	0.950	0.946	0.977
Inverse difference (GLCM)	0.985	0.986	0.995	0.984
Inverse difference moment (GLCM)	0.986	0.985	0.992	0.978
Inverse difference moment normalized (GLCM)	0.948	0.879	0.995	0.953
Inverse difference normalized (GLCM)	0.937	0.899	0.990	0.988
Informational measure of correlation-1 (GLCM)	0.976	0.979	0.995	0.982
Informational measure of correlation-2 (GLCM)	0.981	0.971	0.968	0.980
Inverse variance (GLCM)	0.974	0.972	0.995	0.993
Joint average (GLCM)	0.958	0.924	0.975	0.981
Joint energy (GLCM)	0.980	0.983	0.999	0.999
Joint entropy (GLCM)	0.984	0.984	0.953	0.950
Maximal correlation coefficient (GLCM)	0.916	0.939	0.952	0.963
Maximum probability (GLCM)	0.963	0.985	0.995	0.995
Sum average (GLCM)	0.958	0.924	0.976	0.982
Sum entropy (GLCM)	0.982	0.983	0.991	0.995
Sum squares (GLCM)	0.974	0.966	0.972	0.978
Dependence entropy (GLDM)	0.976	0.982	0.995	0.997
Dependence non-uniformity (GLDM)	0.981	0.972	0.979	0.975
Dependence non-uniformity normalized (GLDM)	0.973	0.976	0.997	0.994
Dependence variance (GLDM)	0.978	0.982	0.975	0.972
Gray-level non-uniformity (GLDM)	0.987	0.987	0.983	0.981
Gray-level variance (GLDM)	0.970	0.965	0.974	0.978
High gray-level emphasis (GLDM)	0.960	0.933	0.981	0.984
Large dependence emphasis (GLDM)	0.982	0.991	0.996	0.992
Large dependence, high gray-level emphasis (GLDM)	0.887	0.865	0.948	0.974
Large dependence low gray-level emphasis (GLDM)	0.961	0.900	0.995	0.982
Low gray-level emphasis (GLDM)	0.942	0.863	1.000	1.000
Small dependence emphasis (GLDM)	0.980	0.982	1.000	0.991
Small dependence, high gray-level emphasis (GLDM)	0.975	0.957	0.989	0.986
Small dependence, low gray-level emphasis (GLDM)	0.894	0.824	0.996	0.999
Gray-level non-uniformity (GLRLM)	0.985	0.988	0.981	0.979
Gray-level non-uniformity normalized (GLRLM)	0.981	0.978	0.995	0.984
Gray-level variance (GLRLM)	0.963	0.960	0.983	0.974
High gray-level run emphasis (GLRLM)	0.959	0.936	0.981	0.985
Long run emphasis (GLRLM)	0.974	0.992	0.995	0.989
Long run high gray-level emphasis (GLRLM)	0.886	0.842	0.970	0.981
Long run low gray-level emphasis (GLRLM)	0.961	0.888	0.794	0.874
Low gray-level run emphasis (GLRLM)	0.942	0.849	0.999	0.999
Run entropy (GLRLM)	0.975	0.978	0.984	0.972
Run length non-uniformity (GLRLM)	0.978	0.970	0.985	0.982
Run length non-uniformity normalized (GLRLM)	0.983	0.986	0.972	1.000
Run percentage (GLRLM)	0.984	0.989	0.998	0.998
Run variance (GLRLM)	0.973	0.992	0.996	0.985
Short run emphasis (GLRLM)	0.982	0.986	0.991	0.980
Short run high gray-level emphasis (GLRLM)	0.965	0.945	0.983	0.985
Short run low gray-level emphasis (GLRLM)	0.934	0.841	0.997	0.983
Gray-level non-uniformity (GLSZM)	0.972	0.979	0.975	0.974
Gray-level non-uniformity normalized (GLSZM)	0.978	0.971	0.973	0.985
Gray-level variance (GLSZM)	0.936	0.933	0.965	0.969
High gray-level zone emphasis (GLSZM)	0.953	0.939	0.979	0.983
Large area emphasis (GLSZM)	0.953	0.977	0.974	0.944
Large area high gray-level emphasis (GLSZM)	0.865	0.941	0.927	0.972
Large area low gray-level emphasis (GLSZM)	0.942	0.872	0.840	0.765
Low gray-level zone emphasis (GLSZM)	0.943	0.796	1.000	1.000
Size zone non-uniformity (GLSZM)	0.927	0.954	0.971	0.962
Size zone non-uniformity normalized (GLSZM)	0.934	0.973	0.989	0.986
Small area emphasis (GLSZM)	0.934	0.974	0.988	1.000
Small area high gray-level emphasis (GLSZM)	0.956	0.947	0.984	0.986
Small area low gray-level emphasis (GLSZM)	0.912	0.742	0.998	0.984
Zone entropy (GLSZM)	0.974	0.973	0.990	0.981
Zone percentage (GLSZM)	0.984	0.982	0.989	0.998
Zone variance (GLSZM)	0.951	0.978	0.992	0.987
Busyness (NGTDM)	0.952	0.948	0.930	0.941
Coarseness (NGTDM)	0.983	0.950	0.974	0.963
Complexity (NGTDM)	0.949	0.925	0.960	0.943
Contrast (NGTDM)	0.975	0.947	0.991	0.986
Strength (NGTDM)	0.868	0.773	0.765	0.889
